# *Arquivos de Neuro-Psiquiatria* , 80 years: part 1 (1943–1962)

**DOI:** 10.1055/s-0045-1804917

**Published:** 2025-03-19

**Authors:** Rodrigo Fagundes da Rosa, Francisco Duque de Paiva Giudice Junior, Francisco de Assis Aquino Gondim

**Affiliations:** 1 Universidade Federal do Ceará, Faculdade de Medicina, Fortaleza CE, Brazil.; 2 Universidade Federal do Ceará, Faculdade de Medicina, Departamento de Medicina Clínica, Fortaleza CE, Brazil.

**Keywords:** Arquivos de Neuro-Psiquiatria, Neurology, History, Periodical

## Abstract

**Background** *Arquivos de Neuro-Psiquiatria* (ANP), the official journal of Academia Brasileira de Neurologia (ABN, Brazilian Academy of Neurology), celebrated 80 years in 2023.

**Objective** To analyze the publication trends, authorship, and editorial patterns of the first 20 volumes of ANP.

**Methods** We analyzed the first 20 volumes of ANP, published from 1943 to 1962. The data were tabulated independently by two blinded researchers and cross-verified.

**Results** Oswaldo Lange was the chief editor, and, from 1943 to 1962, 20 volumes, 79 issues, 2 supplements, and 885 articles were published. We analyzed 509 articles (and excluded non-research papers). We found 905 authors (1.78 authors/article), and only 3.75% were women. Horacio Martins Canelas, Rolando Ângelo Tenuto, and Paulo Pinto Pupo were the most prolific authors. There were 326 papers on neurology, 83 on neurosurgery, 88 on psychiatry, and 12 on basic research. A comparison between the first 10 and second 10 volumes disclosed a significant difference in the fields of the articles: a progressive decrease in papers on psychiatry and an increase in those on neurology and basic science ( *p* = 0.005). There was also a significant decrease in the total number of published articles in the second 10 volumes ( *p* = 0.001), and a higher number of citations per article ( *p* = 0.014), but no difference in the number of pages (mean number in the original articles: 11.9 ± 6.7 pages). Although most articles came from Southeastern Brazil (74%) and were written in Portuguese (84%), 91 were foreign.

**Conclusion** The first 20 volumes marked the establishment of ANP in the post-World War II era. Most papers were written in Portuguese and included international contributions from Egas Moniz, Barraquer-Bordas, Bing, Denny-Brown and Wartenberg, for example.

## INTRODUCTION

Medical journals devoted to Neurology started to flourish at the end of the twentieth century, after the establishment of neurology as a medical subspecialty. However, the boundaries between neurological and psychiatric subjects were not clear. This can be seen in publications such as the *West Riding Lunatic Asylum Medical Reports* , founded in 1871 and considered to be a precursor of *Brain* , the first established neuroscientific journal. 1 *Brain* was founded in 1878 by John Charles Bucknill, David Ferrier, James Crichton-Browne, and John Hughlings Jackson. *Revue Neurologique* , founded by Charcot in 1893, and the first neuroscientific journal in North America, *Archives of Neurology and Psychiatry* (now *JAMA Neurology* ), founded in 1919, further illustrate this earlier trend.

In Brazil, *Arquivos de Neuro-Psiquiatria* (ANP), the official journal of Academia Brasileira de Neurologia (ABN, Brazilian Academy of Neurology), shared similarities and a rich history dating back to its inception in 1943. Established in June of that year by a trio of pioneering neurologists – Oswaldo Lange, Adherbal Tolosa, and Paulino Longo –, the journal has played a pivotal role in the development and advancement of neurology in Brazil. 2

In the decades since its foundation, ANP has been at the forefront of the Brazil's neuroscience research, serving as a vital platform for the dissemination of groundbreaking findings and the exchange of ideas among the medical community. The journal's establishment coincided with the formative years of neurology as a distinct medical discipline in Brazil, a period marked by the foundation of the ABN, in 1962. 3 Over the years, ANP has undergone numerous improvements, including indexing in international databases, the implementation of peer-review processes, and the transition to an open-access model. These advancements have solidified the journal's reputation for academic excellence and fostered the internationalization of Brazilian neuroscience research. The present article, the first in a series of four, analyzes the publication profile of the first 20 volumes of ANP, covering the period within its first 20 years of existence (1943–1962), shedding light on its historical significance and its pivotal role in shaping the field of neurology in Brazil. Part of the current study was reported in abstract form at the XXXI Brazilian Congress of Neurology. 4

## METHODS

We analyzed the first 20 ANP volumes, published from 1943 to 1962. Each article from the internet-based repository was individually reviewed. In addition, all physical editions available at the Professor Jurandir Marães Picanço Health Sciences Library at Universidade Federal do Ceará were also examined. Data from both sources were tabulated by two independent and blinded researchers (RFR and FDPGJ). Subsequently, the two researchers cross-verified the collected data. The following parameters were analyzed in each article: year of publication, number of authors, number of pages, city/state/country of the institution that submitted the article, gender of the first author, and article type (original article, case report, practical note, conferences, update article, technical notes, preliminary notes, historical notes, symposia, doctoral thesis, and habilitation thesis). Articles from the following categories were excluded from the analysis, since they frequently did not list the author/institution: book reviews, magazine reviews, editorials, *errata* , homages, scientific meetings, medical congresses, current events, generalities, obituaries ( *in memoriam* ), lectures, speeches, special, scientific comments, and the ANP's presentation article. We also evaluated the total number of citations from each article as detected by Dimensions AI (Digital Science & Research Solutions Inc., London, United Kingdom), available at the ANP web site. Descriptive statistics was conducted to detail the most important aspects of the publication (mean ± standard deviation [SD]) and the Chi-squared test and the two-tailed *t* -test were used to compare the trends in editorial findings in the first 10 and in the second 10 volumes.

## RESULTS

**Figure 1:**
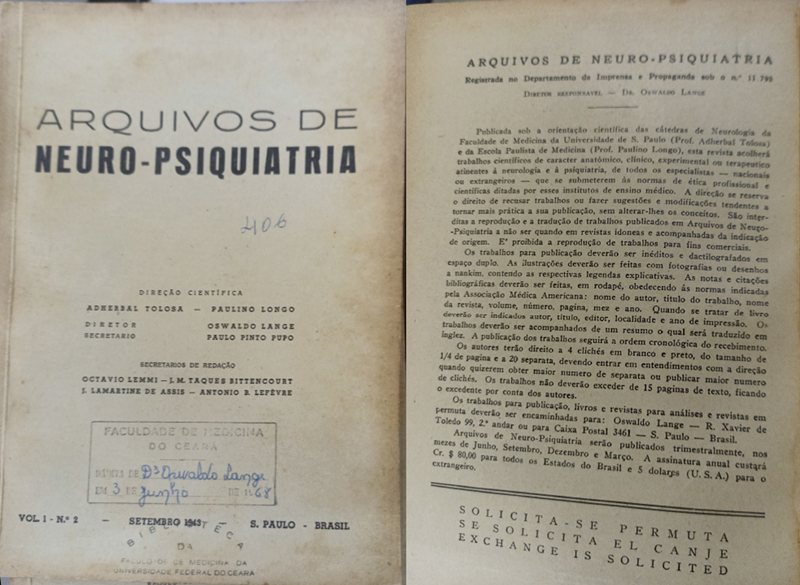
Front and back covers of one of the first editions of the *Arquivos de Neuro-Psiquiatria* (ANP) initially published in 1943, found in the collection of Professor Jurandir Marães Picanço Health Sciences Library at the Universidade Federal do Ceará, in the city of Fortaleza, state of Ceará, Brazil. These editions include information about the first editorial board, as well as a description of the journal and submission instructions.

In the first two decades, the editorial board of the journal changed a few times. As shown in Figure 1 , Professor Oswaldo Lange (from Universidade de São Paulo) served as chief editor from 1943 to 1986, Professors Adherbal Tolosa and Paulino Longo shared the scientific direction, and Paulo Pinto Pupo was the secretary. In June 1947, Professor Horacio Martins Canelas also became general secretary, together with Paulo Pinto Pupo. In December 1956, there were 4 scientific directors: Professors Adherbal Tolosa, Paulino Longo, Paulo Pinto Pupo, and Oswaldo Freitas Julião. From September 1958 to 1962, only the first 2 continued as scientific directors.

Between 1943 and 1962, 79 issues and 2 supplements were published in 20 volumes of the journal. A total of 885 articles of all types were found. During this period, 4 issues were published annually, except in 1943, the inaugural year, in which only 3 issues were published, and in 1954, when 2 additional supplements were published (in a total of 6 issues in that year; thus, there are 2 supplements not available in the ANP's internet-based repository, which were evaluated in paper format from volumes available at Universidade Federal do Ceará's Health Sciences Library).

**Figure 2:**
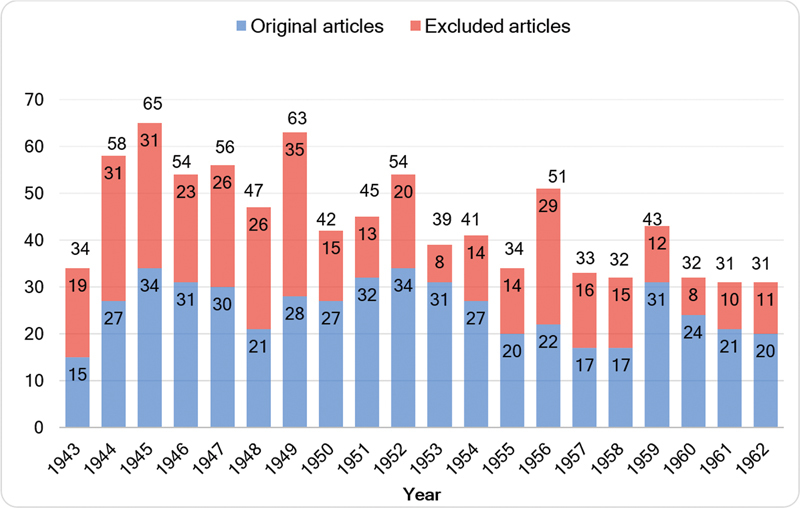
Number of analyzed (blue) and non-analyzed (red) articles published per year from 1943 to 1962, with the total number of articles above each column.

After excluding 376 articles, a total of 509 papers were analyzed ( Figure 2 details the number of analyzed and non-analyzed articles published per year. We identified 905 authors, comprising 1.78 authors per article during the specified period. The articles were then further classified in the following categories: 300 “original articles,” 103 “case reports,” 54 “conferences,” 17 “practical notes,” 13 “preliminary notes,” 9 “updates,” 6 “symposia,” 3 “technical notes,” 1 “historical note,” and 1 report presented at the XIX International Congress of Otoneuro-Ophthalmology. In addition, the journal published two supplements, one containing a doctoral thesis by Rolando Ângelo Tenuto and the other, a habilitation thesis by Roberto Melaragno Filho. Professor Horacio Martins Canelas stands out as the most prolific author in the first 20 volumes, with 24 contributions as main author and 8 as coauthor, in a total of 32 contributions. In addition, Rolando Ângelo Tenuto, with 30 contributions (12 times as main author and 18 times as coauthor), and Paulo Pinto Pupo, with 27 contributions (18 times as main author and 9 times as coauthor), were the second and third most productive authors. Table 1 shows the top ten most productive authors in the first 20 volumes of the journal. Out of the 905 authors identified, only 34 (3.75%) were women. Maria Elisa Bierrenbach Khoury was the first woman to coauthor an ANP article (second edition from 1943). Iracy Doyle stands out as the woman with the highest number of first authorships during this period. 4 Maria I. Valente had 6 coauthorships, standing as the woman with the highest number of publications during the studied period.

**Table 1:** The 10 most prolific authors during the first 20 volumes

N°	Author	Authorships	Coauthorships	Total*
1	Horacio Martins Canelas	24	8	32
2	Rolando Ângelo Tenuto	12	18	30
3	Paulo Pinto Pupo	18	9	27
4	José Lamartine de Assis	18	5	23
5	José Zaclis	13	10	23
6	Aloysio Mattos Pimenta	7	15	22
7	Oswaldo Ricciardi Cruz	8	13	21
8	Antonio B. Lefèvre	15	5	20
9	Antonio Spina-França Netto	15	4	19
10	Roberto Melaragno Filho	13	6	19

Note: *Main authorships + coauthorships.

According to the main study fields, there were 326 neurology articles, 83 neurosurgery articles, 88 psychiatry articles, and 12 basic research papers. Table 2 further details the main subspecialties of the neurology articles. Neuro-Infection was the most popular subject of the original papers, followed by cerebrovascular, neuro-oncology, neuromuscular, and cerebrospinal fluid studies. The Chi-squared test revealed a significant change in the distribution of the articles by main subject ( *p* = 0.005) when one compares the distribution in the first versus the second 10 volumes: neurology – 172/279 versus 154/230; neurosurgery – 46/279 versus 37/230; psychiatry – 59/279 versus 29/230; and basic research – 2/279 versus 10/230). As the data show, this change is mostly due to a decreased percentage of psychiatry articles and an increased percentage of basic science original articles.

**Table 2:** Shows the main subjects covered in the articles analyzed, categorized as ‘Neurology’

Main subjects	Number of articles
Behavioral neurology	6
Cerebrospinal fluid	23
Epilepsy	14
Headache	3
Myelopathy	11
Movement disorders	9
Neuro-Infection	48
Neurogenetics	14
Neuroimmunology	10
Neuromuscular	28
Neuroncology	30
Neuropediatrics	21
Neurophysiology	17
Neurovascular	38
Others	53
Peripheral Neuropathy	1

**Figure 3:**
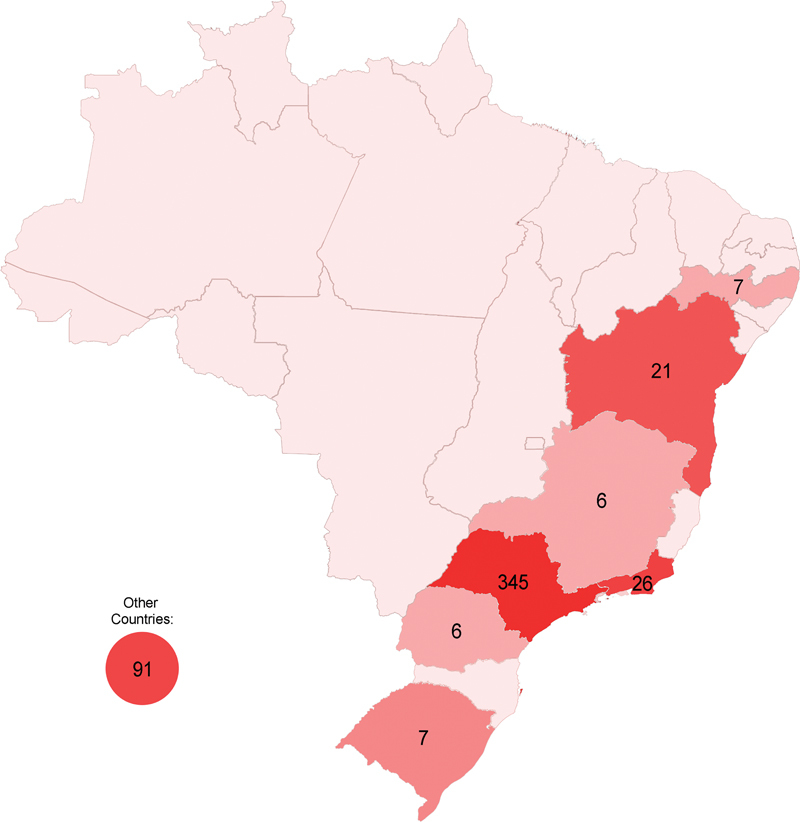
Map of Brazil in 1962 with the number of articles in each state and the number of international articles.

Figure 3 shows the geographical distribution of publications from Brazil. The origin of most articles was Southeastern Brazil (74%). Only 8% were from Northeastern, MidWestern or Southern Brazilian states, and no original articles were from Northern Brazil (Amazon area). A total of 91 foreign articles came from the following countries: France (17), Argentina (16), the United States of America (13), Spain (11), Portugal (9), Denmark (5), Germany (4), Bolivia (3), Norway (2), Switzerland (2), England (2), Peru (1), Greece (1), Uruguay (1), Canada (1), Belgium (1), Netherlands (1), and Italy (1). Most of the articles (84%) were published in Portuguese. A few articles were published in the following languages: English (5.1%), French (5.5%), Spanish (5.3%), and Italian (0.1%).

**Figure 4:**
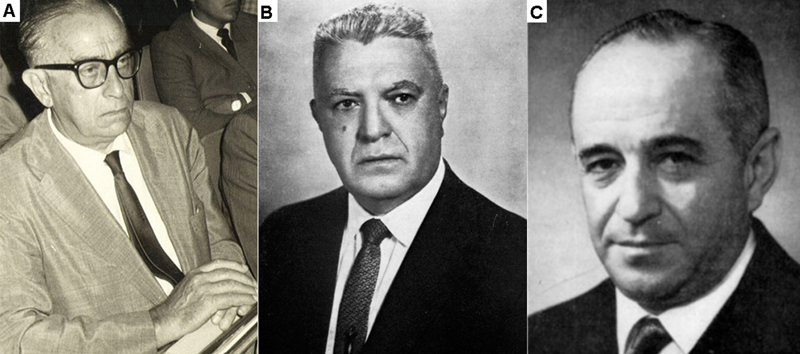
The founding trio of the ANP. ( **A** ) Oswaldo Lange. ( **B** ) Adherbal Tolosa. ( **C** ) Paulino Watt Longo.

Figure 2 details the total number of original articles and the total number of all article subtypes published per year from 1943 to 1962. The mean number of all article subtypes and of all original articles published per year was of 44.3 ± 11.3 and 25.5 ± 6 respectively. There was no significant difference in the mean number of original articles published in the first 10 volumes (27.9 ± 6) and in the second 10 volumes (23 ± 5.2) ( *p* > 0.05). However, there was a significant decrease in the mean total number of published articles from the first 10 (51.8 ± 9.7) to the second 10 volumes (36.8 ± 7.2), which were published from 1953 to 1962 ( *p* = 0.001). The mean number of pages of the original articles analyzed during the period was of 11.9 ± 6.7, ranging from 1 to 92 pages. The mean number of pages in the first 10 volumes (12 ± 1.5) was similar in comparison with the second 10 years (12.8 ± 2.4) ( *p* > 0.05). Over the course of 20 years, the mean total number of citations per article was of 2.64 ± 5.55. Figure 4 details the mean ± SD total number of citations per article for each year. There was a significant increase in the number of citations per article when one compares the first 10 volumes with the last 10 volumes: 1.94 ± 4.2 and 3.72 ± 6.4 respectively ( *p* = 0.014). Figure 4 also details the 10 articles with the highest number of citations throughout the first 20 volumes.

## DISCUSSION

The first scientific publications in the field of neurology in Brazil followed the same trend as those from Europe and the United States, where the boundaries between neurology and psychiatry were not clearly established. *Arquivos Brasileiros de Psiquiatria, Neurologia e Ciências Afins* , founded in 1905 by Juliano Moreira at Hospício Nacional de Alienados and discontinued in 1918, is considered the first Brazilian scientific journal dedicated to neurological and psychiatric subjects. 5 In 1928, Americo Ricaldoni organized the publication of *Anales del Instituto de Neurología* in Montevideo, Uruguay. 6 In Argentina, *Revista Neurológica de Buenos Aires* (1936) and *Folia Neurobiológica Argentina* (1939) were founded and stand among the pioneering neurology journals in Latin America. 7 Similarly, *Revista Neurobiologia* , founded by Ulysses Pernambucano in 1938, was one of the first publications in Brazil following a similar trend. 8

The first 20 volumes of ANP established it as a vital platform to disseminate knowledge, with significant contributions from Brazilian researchers. Geographically, there was a significant concentration of publications from the Southeastern Region of Brazil, mainly from the state of São Paulo, the journal's birthplace and the operating location of its founders ( Figure 3 ). The initial contribution of female authors was limited, not reaching 5% of the articles, but statistics from the Brazilian Federal Medicine Council 9 describes that women comprised 13% of the physicians in the 1950s and 1960s, with a high likelihood of an even lower percentage of female physicians in neurology, neurosurgery and psychiatry during this period.

Additionally, in the first 20 years, it is important to highlight the presence of publications from 15 other countries, including Argentina, France, Spain, and the United States. This international representation demonstrates the journal's commitment to fostering a diverse global scholarly community. In this sense, although most of the papers were published in Portuguese (84%), a significant percentage was published in several other languages, in an attempt to attract the attention of established neurologists from different countries. Despite the initial dominance of certain regions in terms of publication productivity and visibility, the journal demonstrated a commitment to address regional and global scientific needs during its formative years, establishing itself as a platform for the exchange of ideas and the dissemination of cutting-edge neuroscience research. This endeavor not only reflects the journal's early efforts to promote inclusiveness in scientific discourse but also underscores the importance of collaborative research across borders, which was essential to advance the field of neuroscience and psychiatric studies globally.

**Figure 5:**
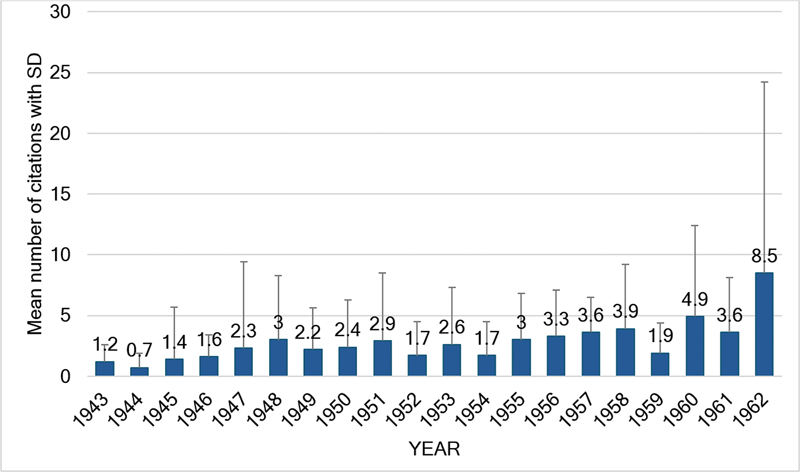
Details of the mean number of citations in each of the 20 volumes analyzed.

In the introductory article of the journal, Tolosa and Longo summarize some of the objectives of the publication: “The delay in the publication of papers or their publication in non-specialized journals is undoubtedly a serious inconvenience, the consequences of which have been felt mainly by the two schools of clinical neurology in São Paulo: Universidade de São Paulo and Escola Paulista de Medicina.” This statement highlights the hurdles experienced by Brazilian neurologists of that time to reach international journals, especially due to language barriers (most of the neurology journals were either written in English or French). In this sense, a scientific journal in Portuguese helped establish the first generation of neurology scholars in Brazil. Figure 5 and Table 3 show the foundation trio of ANP. In view of the figures reported up to this point, it is worth highlighting the importance of this trio for the development of Brazilian neurology resulting from the vitality of the tree planted by Enjolras Vampré. 10

**Table 3:** List of the 10 most cited articles in the first 20 volumes, with the total number of citations

N°	Article	Citations
1	Canelas (1962) | Neurocisticercose: incidência, diagnóstico e formas clínicas 19	70
2	Lacaz and Bittencourt (1947) | Micoses do sistema nervoso 20	32
3	Lemmi and Pimenta (1960) | Granuloma paracoccidióidico cerebral: a propósito de um caso operado 21	29
4	Canelas et al. (1951) | Blastomicose do sistema nervoso 22	26
5	Gama et al. (1945) | Esquistossomose medular: Granulomas produzidos por ovos de Schistosoma Mansoni comprimindo a medula, epicone, cone e cauda eqüina 23	25
6	Brotto (1947) | Aspectos neurológicos da cisticercose 24	24
7	Ritter (1948) | Tumor cerebral granulomatoso por paracoccidióide: A propósito de dois casos operados 25	24
8	Finkel (1962) | A forma pseudomiopática tardia da atrofia muscular progressiva heredo-familial 17	22
9	Nussenzveig et al. (1953) | Acidentes vasculares cerebrais embólicos na cardiopatia chagásica crônica 26	20
10	Spina-França (1960) | Electroforese em papel das proteínas do líquido cefalorraquidiano: IV. valores normais 27	20

Professor Oswaldo Lange was the pillar and editor of the journal from its foundation in 1943 until his death in 1986. A disciple of Vampré, medical doctor, physician and director of clinical practice at Universidade de São Paulo, Lange conceived and coordinated the journal in an exemplary manner, with an emphasis on regularity and periodicity of publication. In 1944, Dr. Oswaldo Lange had direct contact with Morris Fishbein, editor of the *Journal of the American Medical Association* (JAMA), from which he developed and perfected the editorial policy developed during his time at ANP. Dr. Lange was also a key figure in the early internationalization of the journal, as evidenced by the choice of the journal as the vehicle for a tribute to Prof. Dr. Luiz Barraquer Ferré, a cornerstone of Spanish neurology, in 1952. 11 This special issue included papers from world neurological leaders such as Paul Robert Bing, Derek Denny-Brown, and Robert Wartenberg. Professor Adherbal Tolosa was responsible for the scientific direction of the ANP throughout the period studied, and it is worth highlighting his importance in the development of Brazilian neurology. Dr. Tolosa took over the Chair of Neurology at Universidade de São Paulo after the death of his mentor, professor Vampre in 1938. Dr. Adherbal Tolosa was the director and an author of the journal since its inception, and in 1962. With the creation of ABN, he was elected president of its first board of directors. 12 In 1938, Dr. Paulino Watt Longo took over the Chair of Clinical Neurology at Escola Paulista de Medicina and, in 1943, together with Professor Tolosa, they formed the first Scientific Council of ANP. 13

When one compares the first 10 and the second 10 volumes, there was a decrease in the percentage of psychiatry articles and an increase in the proportion of basic science and neurology papers. This trend highlights the subsequent separation between neurology and psychiatry subjects. Among the earlier topics covered in the journal's articles, some of the most curious were related to World War II (WWII), such as “Higiene mental de guerra” 14 (“War mental hygiene”), a book review on the psychological aspects of the Nazi society, some neurosurgical procedures adopted during the conflict, 15 as well as a psychiatry article entitled “Reação delirante induzida; estudo de epidemia mental coletiva” (“Induced delusional reaction; study of a collective mental epidemic”), which reported a family of young mediums from the Brazilian countryside who, in addition to promoting supposed spiritual cures, prophesied the end of the war and the death of Hitler. 16 Most of the articles written by Brazilian authors were the first descriptions of several neurological conditions in Brazil, and the article by Nunjo Finkel, 17 published in 1962, stands as the first clinical description of large kindreds from Southeastern Brazil affected by familial ALS later linked to mutations in the *VAPB* gene.

Table 2 also highlights the percentage of papers regarding the different neurological subspecialties. Neuro-Infection was the most popular subject of the original papers, followed by cerebrovascular, neuro-oncology, neuromuscular, and cerebrospinal fluid studies. However, the diversity of articles was such that the journal covered all major neurological subspecialties, in addition to psychiatry and neurosurgical subjects. Several papers from this period dealt with lobotomies, an important topic in the boundaries of neurology, neurosurgery, and psychiatry. Egaz Moniz, the Nobel Prize winner for lobotomy, did contribute with an article in the subject of cerebrovascular disease and neuroimaging, not related to lobotomy. 18 Throughout the first 20 years, there was a significant decrease in the total number of articles in the second set of 10 volumes ( *p* < 0.05), but not in the total number of original articles, showing that the editors further attempted to decrease the number of non-research contributions. The mean total number of pages remained stable over the first 20 years (11.9 ± 6.7; range: 1–92 pages), but this article length was common from the 1940s to 1960s, as we anticipate a significant decrease in the future comparison over the next 60 years. As can be seen in Figure 4 , the total number of citations detected by Dimensions AI also significantly ( *p* < 0.05) increased in the second set of 10 volumes, highlighting the progressive international impact of the journal. Not surprisingly, Table 3 also details that most of the articles with the highest number of citations from this period dealt with neuro-infection diseases.

In summary, the first 20 volumes marked the establishment of ANP in the post-WWII era. Most papers were written in Portuguese but were also available in other languages (mostly French and English) from 15 other countries, including publications from Nobel Prize winner Antonio Caetano de Abreu (Egaz Moniz) and worldwide famous neurologists such as Luís Barraquer-Bordas, Paul Robert Bing, Derek Denny-Brown, and Robert Wartenberg.
